# Gastroenterologists Adherence to Tumor Necrosis Factor Antagonist Combination Therapy in Inflammatory Bowel Disease

**DOI:** 10.3389/fmed.2021.725512

**Published:** 2021-09-29

**Authors:** Israa Abdullah, Ghadeer Alhendi, Anwar Alhadab, Hajer Alasfour, Mohammad Shehab

**Affiliations:** ^1^Department of Pharmacy Practice, Faculty of Pharmacy, Kuwait University, Kuwait City, Kuwait; ^2^Division of Gastroenterology, Department of Internal Medicine, Mubarak Alkabeer University Hospital, Kuwait University, Kuwait City, Kuwait

**Keywords:** IBD, anti-TNF, combination, gastroenterologist, age, interest

## Abstract

**Introduction:** Tumor necrosis factor antagonists (anti-TNF) therapies are used for the management of moderate to severe inflammatory bowel disease (IBD). Anti-TNF combination therapy, with immunomodulators, has been shown to reduce immunogenicity, especially for infliximab, improve treatment success rate and patient outcomes. We evaluated factors associated with gastroenterologists adherence to anti-TNF combination therapy.

**Methods:** A retrospective cohort study was performed to evaluate the adherence of gastroenterologists (*n* = 14), at an inflammatory bowel disease center, to anti-TNF combination therapy. Records of patients who received Infliximab (*n* = 137) or adalimumab (*n* = 152) were obtained and their ordering physicians' data was analyzed. Gastroenterologists were divided into six groups according to their age and interest in IBD. The baseline characteristics of their patients were also obtained.

**Results:** The proportion of patients on combination therapy in the young gastroenterologists group was higher than those in the senior gastroenterologists group for both infliximab (83.2 vs. 55.6%, respectively, *P* < 0.001) and adalimumab (59 vs. 30.8%, respectively, *P* < 0.001). Gastroenterologists with interest in inflammatory bowel disease (IBD interest group) had also more proportion of patients on adalimumab combination therapy compared to gastroenterologists with no interest in IBD (non-IBD interest group) (61.7 vs. 35.2%, respectively, *P* < 0.001). Gastroenterologists who were both young and have interest in IBD had more proportion of patients on combination therapy than those who were senior or had no interest in IBD for both infliximab (89.4 vs. 63.4%, respectively, *P* < 0.001) and adalimumab (75.9 vs. 33%, *P* < 0.001). The IBD interest group was also requesting more antidrug antibody level tests than those in the non-IBD interest group (41.4 vs. 12.3 tests, respectively, *P* < 0.001).

**Conclusion:** Young gastroenterologists are more likely to prescribe anti-TNF infliximab and adalimumab combination therapy than senior gastroenterologists. In addition, gastroenterologists with IBD interest are more likely to prescribe adalimumab combination therapy than gastroenterologists with no IBD interest. Moreover, young gastroenterologists who have interest in IBD are more likely to prescribe both infliximab and adalimumab combination therapy than senior gastroenterologists or those with no IBD interest. In addition, gastroenterologists with IBD interest requested more anti-TNF serum drug concentrations and antidrug antibody level tests than those with no IBD interest.

## Introduction

Tumor necrosis factor antagonists (anti-TNF) therapies are considered to be an effective treatment for moderate-to-severe inflammatory bowel disease (IBD) (1–4). However, about one-third of patients treated with anti-TNF therapies may experience primary non-response (PNR), manifested by lack of response during induction therapy (5, 6). Moreover, about half of patients with initial response may experience secondary loss of response (SLR) to anti-TNF, by losing treatment effect during the maintenance of remission (6). Immunogenicity, the formation of antidrug antibodies, is known as one of the most common causes of loss of response to anti-TNF (6, 7). The concurrent administration of an immunomodulator with an anti-TNF, combination therapy, has been associated with improvement in the pharmacokinetics of anti-TNF, by decreasing antidrug antibodies and increasing serum drug concentrations, that potentially results in higher rates of clinical remission and lower rates of immunogenicity.

Based on the available evidence (SONIC and UC-SUCCESS trials), both American Gastroenterology Association (AGA) and European Crohn's and Colitis Organization (ECCO) guidelines support the use of infliximab combination therapy over infliximab monotherapy, in inducing and maintaining clinical remission in patients with moderate to severe IBD (1, 3, 8, 9). Whereas, for adalimumab, ECCO guidelines suggest against the use of combination therapy over monotherapy in patients with Crohn's disease (CD) (3). This recommendation was based on DIAMOND trial and two meta-analyses (Kopylov et al. and Chalhoub et al.), which showed that adalimumab combination therapy is associated with limited impact on maintenance of clinical remission or response (10–12). While AGA guidelines support the use of adalimumab combination therapy over monotherapy, especially for patients who have developed loss of response to anti-TNF (1, 2). A recent study done by Roblin et al., showed that combination therapy can result in better clinical outcomes without clinical failure or unfavorable pharmacokinetics at 24 months in patients with IBD who experienced an immune-mediated loss of response with first anti-TNF (13). Moreover, Kuwait local health authority allows physicians to follow any international recognized guidelines, commonly practiced ECCO or AGA guidelines, in regard to IBD management. We did this study to evaluate the adherence of our gastroenterologists to anti-TNF combination therapy and understand factors that can be associated with their adherence.

## Materials and Methods

A retrospective cohort study was conducted at Mubarak Al-Kabeer Hospital, an inflammatory bowel disease center, to measure gastroenterologists adherence to anti-TNF combination therapy in inflammatory bowel disease (IBD). Infliximab and adalimumab prescription records along with infliximab and adalimumab antidrug antibody levels were collected. The data was collected retrospectively using patients' electronic medical records from July 22nd, 2018 until February 7th, 2020.

In addition, C-reactive protein (CRP), serum albumin levels and stool fecal calprotectin levels that were performed within 7 days of serum drug concentration/antidrug antibody collections, were also obtained. Trough serum drug concentration was performed either reactively (e.g., treatment failure) or proactively (e.g., at week 14 for infliximab) in all patients' cohort, who were studied, according to individual gastroenterologist practice. Moreover, patients' age, body mass index (BMI), extent of the disease, and steroid use were also collected.

This study was performed and reported in accordance with Strengthening the Reporting of Observational Studies in Epidemiology (STROBE) guidelines (14). Ethical approval was obtained by the standing committee for coordination of health and medical research at the Ministry of Health in Kuwait (IRB 2020/1410).

### Study Definitions

Patients on anti-TNF combination therapy are those who received adalimumab or infliximab, with an immunomodulator such as azathioprine, 6-mercaptopurine or methotrexate. Patients who received adalimumab or infliximab alone were considered to be on monotherapy.

We divided our analysis into 6 groups. Gastroenterologists who have interest in inflammatory bowel disease (IBD) were classified as IBD interest group (group A) while those who had no interest in IBD were classified as non-IBD interest group (group B). Moreover, gastroenterologists who were younger than 45 years of age were classified as young gastroenterologists (group C) and those who were 45 years or older were classified as senior gastroenterologists (group D). In addition, gastroenterologists who were both younger than 45 years old and had interest in IBD were called young IBD group (group E) while gastroenterologists who were either more than 45 years of age and have IBD interest or had no interest in IBD regardless of their age were called non-young IBD group (group F).

### Outcomes

The primary outcome was the association between gastroenterologist age (≥45, or, <45) or inflammatory bowel disease (IBD) interest and the proportion of patients on combination therapy vs. monotherapy for both infliximab and adalimumab. Moreover, the combined effect of age and interest in IBD on the percentage of patients on combination therapy for infliximab or adalimumab was examined.

### Statistical Analysis

The statistical analysis was performed with the SPSS Statistics Version 27.0. Armonk, NY: IBM Corp. Chi square test was used to examine the association between the categorical groups. We tested the association between gastroenterologists' interest and age, at Haya Alhabib Gastroenterology Center, and the use of anti-TNF combination therapy compared to monotherapy.

*T*-test analysis was conducted for parametric data to calculate the correlation between the number of tests performed among variables in the categorical groups with a 95% confidence interval for the mean difference between the groups. Descriptive analyses were performed to calculate frequencies and proportions within the groups. Two-tailed statistical significance level was used throughout the analysis and set to α = 0.05 for all associations.

## Results

### Demographics

The study included 14 gastroenterologists at Haya Alhabib Gastroenterology Center, of which, 4 (28.5%) have interest in inflammatory bowel disease (IBD). Of all the gastroenterologists, 5 (35.7%) of them were equal or above the age of 45 years at the end of the study period. Only one gastroenterologist was above 45 years old and has interest in IBD. The total number of patients included in the study is 289, of which 137 (47.4%) patients were on infliximab [33 (24%) monotherapy and 104 (75.9%) combination therapy] and 152 (52.6%) patients on adalimumab [77 (50.6%) monotherapy and 75 (49.3%) combination therapy] (Table 1). In all groups, the median disease duration was 10 years, and the median anti-TNF therapy use was 4 years (Table 2).

**Table 1:** Sample description.

**Variable**	**Gastroenterologists characteristics**	**Total patients in the study sample**	***P-*value**
	**(*n* = 14)**	**(*n* = 289)**	
Age group (*n*%)			
≥45	5 (35.7%)	88 (30.4%)	
<45	9 (64.28)	201 (69.5%)	
Subspecialty (*n*%)			
Interest in IBD	4 (28.5%)	166 (57.4%)	
No interest in IBD	10 (71.4%)	123 (42.5%)	
Anti-TNF drug (*n*%)	Young gastroenterologists	Senior gastroenterologists	
Infliximab (*n* = 137)			
Monotherapy (*n* = 33)	17 (16.8%)	16 (44.4%)	
Combination therapy (*n*-104)	84 (83.2%)	20 (55.6%)	*P*-value^†^ < 0.001
Total^*^	101 (100%)	36 (100%)	
Adalimumab (*n* = 152)			
Monotherapy (*n* = 77)	41 (41%)	59 (59%)	
Combination therapy (*n* = 75)	77 (50.7%)	16 (30.8%)	*P*-value^†^ < 0.001
Total^*^	100 (100%)	52 (100%)	
Anti-TNF drug (*n*%)	IBD interest	No IBD interest	
Infliximab			
Monotherapy	17 (20%)	16 (30.7%)	
Combination therapy	68 (80%)	36 (69.2%)	*P*-value^†^ = 0.15
Total^*^	85 (100%)	52 (100%)	
Adalimumab			
Monotherapy	31 (38.2%)	46 (64.7%)	
Combination therapy	50 (61.7%)	25 (35.2%)	*P*-value^†^ < 0.001
Total^*^	81	71	

*IBD, Inflammatory bowel disease*.

* *Total number of patients in the group*.

† *Chi-square test*.

**Table 2:** Patient characteristics of each group.

	**Group A**	**Group B**	**Group C**	**Group D**	**Group E**	**Group F**
	***n* = 166**	***n* = 123**	***n* = 201**	***n* = 88**	***n* = 124**	***n* = 165**
Age^*^ (years)						
Mean	29.0	31.5	30.0	30.0	29.3	30.7
Sex, *n* (%)						
Male	88 (53.0%)	67 (54.5%)	108 (53.7%)	47 (53.4%)	69 (55.6%)	86 (52.1%)
Female	78 (47.0%)	56 (45.5%)	93 (46.2%)	41 (46.6%)	55 (44.4%)	79 (47.9%)
Body mass index (BMI)						
Median	23.7	23.9	23.7	23.6	23.8	23.8
Disease extent, *n* (%)						
Ulcerative colitis (UC)	74	55	90	40	55	74
E1: ulcerative proctitis	7 (9.5%)	5 (9.1%)	9 (10.0%)	3 (7.5%)	5 (9.1%)	7 (9.5%)
E2: left sided colitis	22 (29.7%)	16 (29.1%)	27 (30.0%)	12 (30.0%)	17 (30.9%)	22 (29.7%)
E3: extensive colitis	45 (60.8%)	34 (61.8%)	54 (60.0%)	25 (62.5%)	33 (60.0%)	45 (60.8%)
Crohn's disease (CD)	92	68	111	48	69	91
L1: ileal	41 (44.6%)	30 (44.1%)	50 (45.0%)	22 (45.8%)	31 (44.9%)	41 (45.1%)
L2: colonic	9 (9.8%)	7 (10.3%)	11 (9.9%)	5 (10.4%)	7 (10.1%)	10 (10.9%)
L3: ileocolonic	37 (40.2%)	27 (39.7%)	45 (40.5%)	19 (39.6%)	28 (40.6%)	36 (39.6%)
L4: upper gastrointestinal	5 (5.4%)	4 (5.9%)	5 (4.5%)	2 (4.2%)	3 (4.3%)	4 (4.4%)
B1: inflammatory	41 (44.6%)	31 (45.6%)	50 (45.0%)	21 (43.8%)	31 (44.9%)	42 (46.1%)
B2: stricturing	23 (25.0%)	17 (25.0%)	28 (25.2%)	12 (25.0%)	17 (24.6%)	22 (24.2%)
B3: penetrating	28 (30.4%)	20 (29.4%)	33 (29.7%)	15 (31.3%)	21 (30.4%)	27 (29.7%)
Median disease duration (years)	10.2	10.7	10.2	10.1	10.2	10.2
Median anti-TNF therapy duration (years)	4.1	4.6	4.1	4.3	4.1	4.2
CRP, mg/L (median)	6.1	11.3	7.6	7.0	6.1	9.5
Albumin, g/L (median)	40.0	40.0	40.0	40.0	40.0	40.0
Median stool fecal calprotectin ug/g	112.0	124.0	111.0	115.0	110.0	126.0
Steroid use, *n* (%)	16 (9.6%)	15 (12.1%)	26 (12.3%)	5 (5.6%)	15 (12.1%)	16 (9.7%)
Anti-infliximab antibody serum levels, (AU/ml)						
Median	10.7	25.7	13.3	15.3	9.3	17.9
Anti-adalimumab antibody serum levels, (AU/ml)						
Median	7.8	7.4	8.2	6.7	8.0	7.3

*Group A: IBD interest group, group B: non-IBD interest group, group C: young gastroenterologists, group D: senior gastroenterologists, group E: young IBD group, group F: non-young IBD group*.

* *Age at the time of sampling*.

### Outcomes

**Figure 1:**
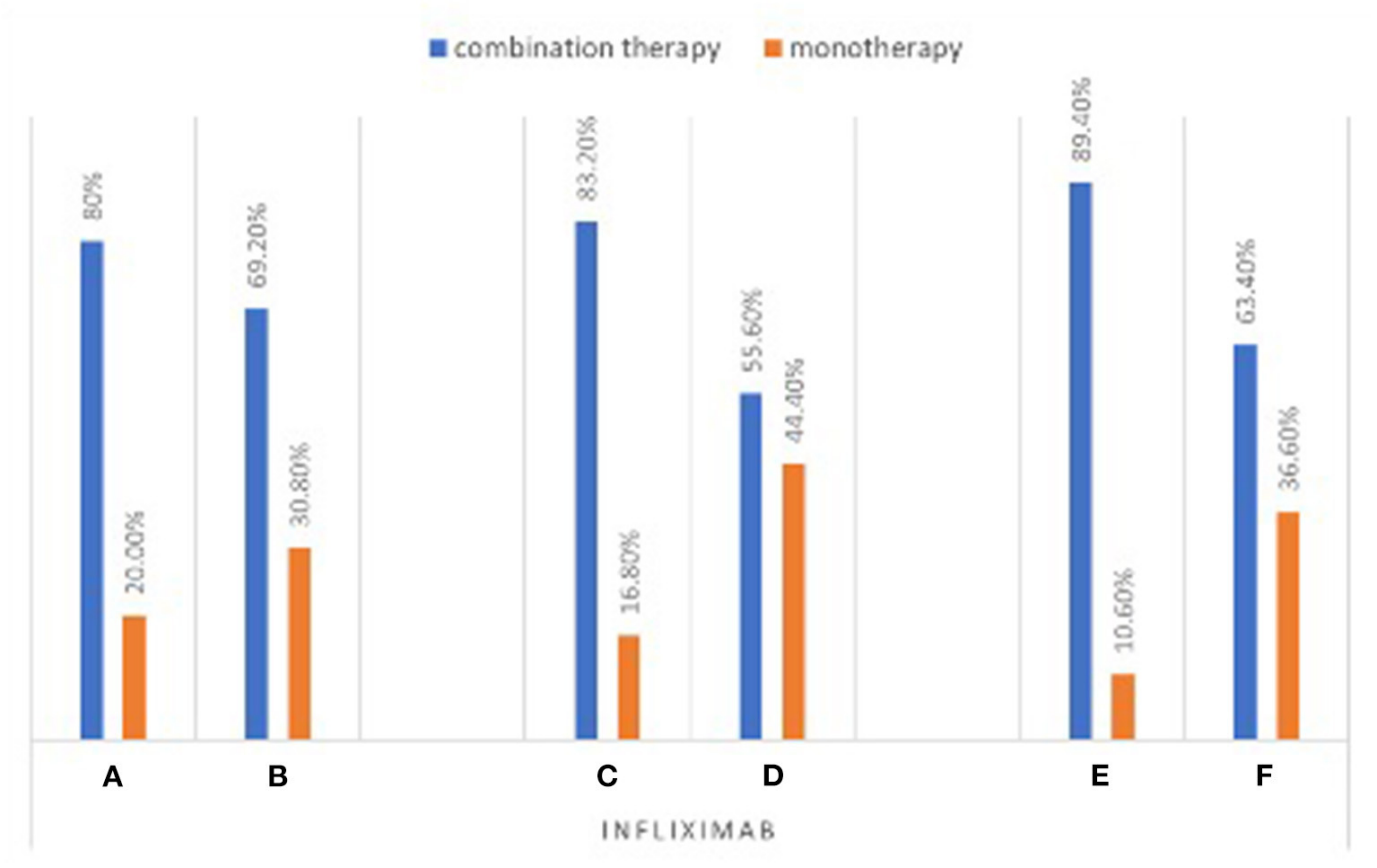
Percentage of patients on infliximab combination therapy stratified by test groups.

**Figure 2:**
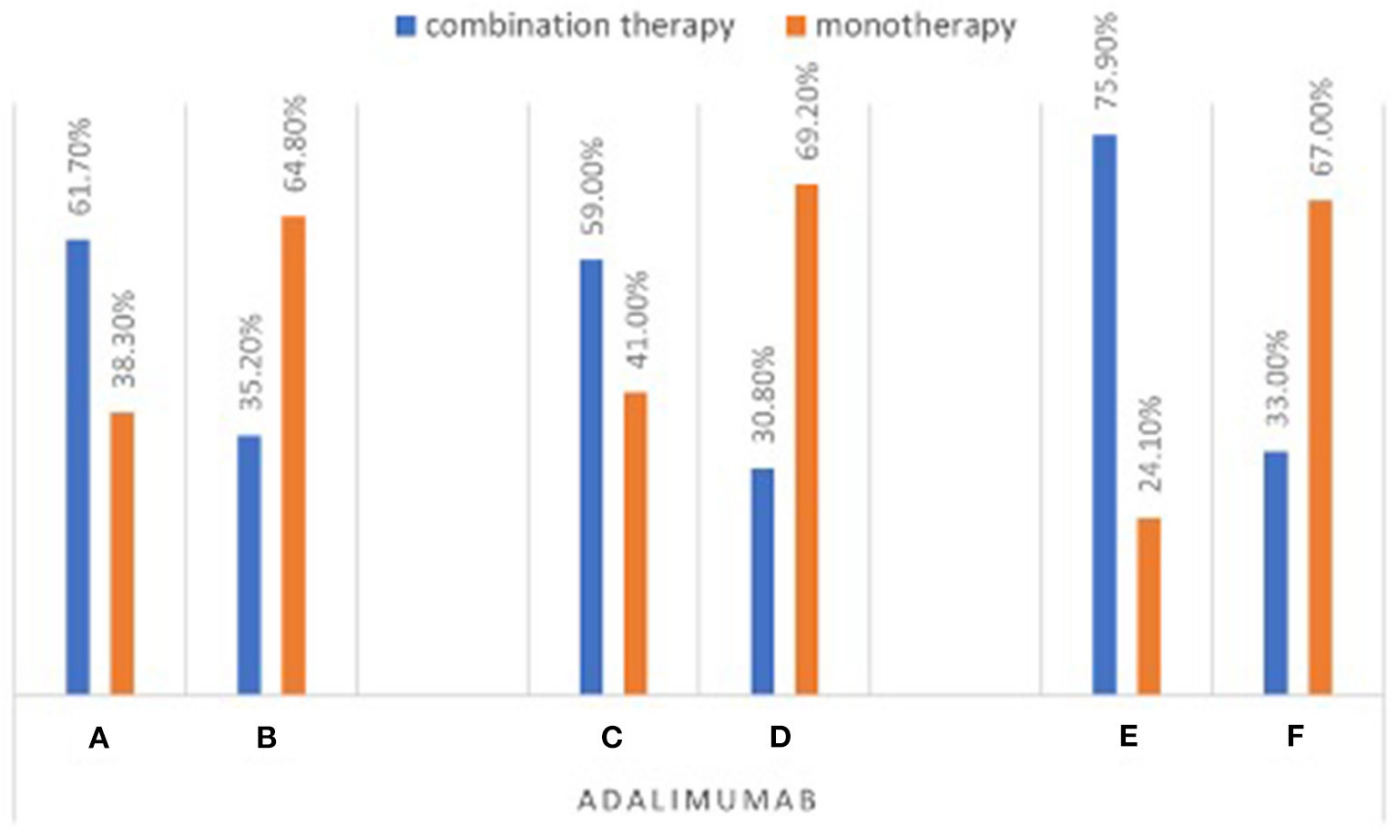
Percentage of patients on adalimumab combination therapy stratified by test groups.

Among patients on infliximab, 83.2% of the patients were on combination therapy in the young gastroenterologists' group (group C) compared to 55.6% in the senior gastroenterologists group (group D) (*P* < 0.001; Figure 1; Supplementary Table 1). Similarly, more patients were found to be on adalimumab combination therapy in the young gastroenterologists' group (group C) than those in the senior gastroenterologists' group (group D) (59, 30.8%, respectively) (*P* < 0.001; Figure 2; Supplementary Table 2). In addition, 80% of patients in the IBD interest group (group A) were on infliximab combination therapy compared to 69.2% of patients in the non-IBD interest group (group B). However, the difference between the two groups was not statistically significant (*P* = 0.153; Figure 1; Supplementary Table 3). Conversely, 61.7% of patients on adalimumab therapy in the IBD interest group (group A) were on combination therapy as opposed to 35.2% of patients in the non-IBD interest group (group B) (*P* < 0.001; Figure 2; Supplementary Table 4).

In the young IBD group (group E) 89.4% of the patients were on infliximab combination therapy compared to 63.4% of patients in the non-young IBD group (group F) (*P* < 0.001; Figure 1). Likewise, 75.9% of patients in the young IBD group (group E) were on adalimumab combination therapy compared to 33% of patients in the non-young IBD group (group F) (*P* < 0.001; Figure 2).

**Figure 3:**
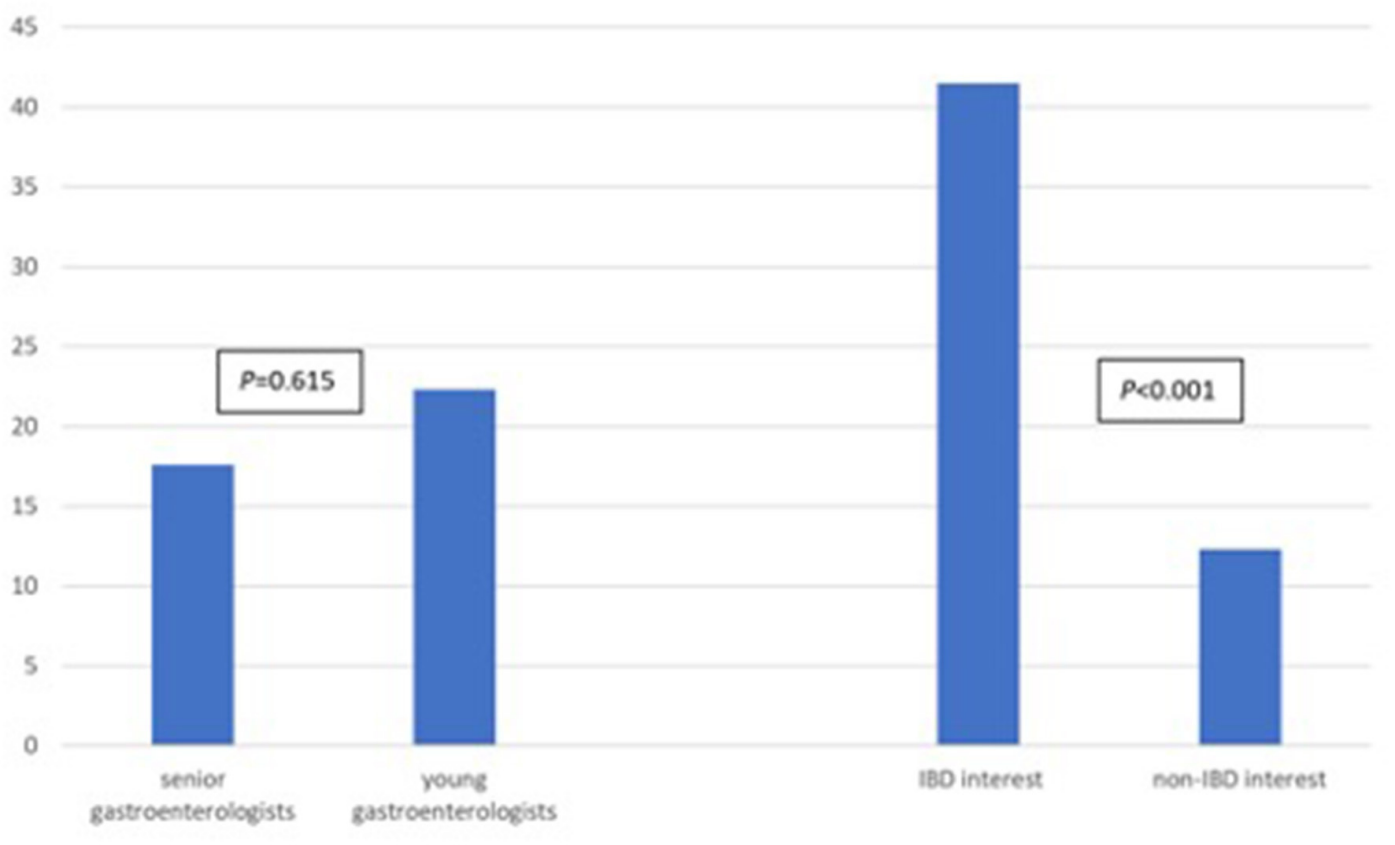
Mean number of tests requested gastroenterologists in the senior vs. young group and in the IBD-interest vs. non-IBD interest group.

Regarding anti-TNF serum drug concentrations and antidrug antibody tests, gastroenterologists in the IBD interest group (group A) requested an average of 41.4 tests while gastroenterologists in the non-IBD interest group (group B) requested an average of 12.3 tests (*P* < 0.001, 95% CI 20.1–40.4; Figure 3; Table 3; Supplementary Table 5). In addition, young gastroenterologists group (group C) requested an average of 22.3 tests compared to an average of 17.6 tests by senio gastroenterologists group (group D) (*P* = 0.615; Figure 3; Table 3).

**Table 3:** *t*-Test, average number of anti-TNF serum drug concentrations and antidrug antibody level tests and requested by young gastroenterologists vs. senior gastroenterologists and in the IBD interest group vs. non-IBD interest group.

	**Senior gastroenterologists**	**Young gastroenterologists**	***P*-value**
Mean number of tests	17.6	22.3	0.615
	**IBD interest**	**non-IBD interest**	
Mean number of tests	41.5	12.3	<0.001

## Discussion

A retrospective cohort study which included 14 gastroenterologists at Haya Alhabib Gastroenterology Center, was conducted to assess the adherence to tumor necrosis factor antagonist (anti-TNF) combination therapy in patients with IBD. Two main factors were found to influence the gastroenterologists' adherence to combination therapy, which were their age and interest in IBD. The study showed that the age of the treating gastroenterologist may have some influence, as the gastroenterologists aged 45 or less, appeared to be more adherent to the combination therapy.

The second factor that played a role in the commitment to combination therapy was the gastroenterologist's interest in IBD, which was observed more with IBD subspecialized gastroenterologists, significantly with adalimumab. Reactive drug monitoring, which is the evaluation of serum drug concentration and antidrug antibody level in active or poorly controlled disease, is commonly performed more frequently by IBD subspecialized gastroenterologists. An Italian multicenter prospective observational study by Scribanoet et al. reported that IBD subspecialized gastroenterologists had a greater adherence to combination therapy for patients with ulcerative colitis (UC) flare, when compared to general gastroenterologists (15). Also, there is a survey study by Grossberg et al., included 606 physicians in the United States of America (USA), showed that academic gastroenterologists and physicians who were seeing more patients with IBD, have utilized therapeutic drug monitoring (TDM) reactively more frequently (16).

TDM is performed to guide the use of anti-TNF by assessing the drug serum concentrations and antidrug antibodies levels. It can be performed at any point of therapy, whether as reactive monitoring, or as routine proactive monitoring provided to patients in remission (17). There is currently insufficient evidence to recommend the routine use of proactive TDM to improve clinical outcomes when compared to routine care for IBD patients in clinical remission under anti-TNF treatment (17). This is based on 2 RCTs, by Vande Casteele et al. and D'Haens et al., with a total of 359 patients treated with infliximab in combination with an immunomodulator, had shown no difference in clinical remission between clinically-based dosing and concentration-based dosing (proactive TDM) groups. However, the concentration-based dosing groups had fewer relapses during follow-up (18, 19). A recent retrospective study, by Papamichael et al., showed that the proactive TDM of infliximab was associated with more favorable therapeutic outcomes and fewer IBD-related hospitalizations and surgery compared to reactive TDM alone (20). Moreover, a systematic review, by Strand et al., emphasized the role of TDM of anti-TNF therapy (21). This might be helpful in guiding the physicians in improving anti-TNF therapy management and achieving better clinical outcomes.

Our study is the first study in Kuwait to evaluate the adherence to anti-TNF combination therapy amongst gastroenterologists. It emphasizes the importance of gastroenterologists' interest in IBD when treating such patients. It also encourages gastroenterologists to be updated and follow recently published IBD guidelines. On the other hand, there are some limitations to our study. It is a retrospective single center study with possible confounders and bias. For example. The lack of data regarding proactive vs. reactive TDM testing, and discontinuation of anti-TNF combination therapy, due to side effects or absence of antibodies, were possible confounders. In addition, clinical and endoscopic remission targets were not assessed. However, objective inflammatory markers (such as serum CRP levels, stool fecal calprotectin levels and steroid use) were obtained. Finally, the small number of physicians in the IBD interest group could have influenced our results.

To sum up, young gastroenterologists (<45 years of age) are more likely to prescribe anti-TNF (infliximab and adalimumab) combination therapy than senior gastroenterologists. In addition, Gastroenterologists with IBD interest are more likely to prescribe adalimumab combination therapy than gastroenterologists with no IBD interest. However, no significant difference was found for prescribing infliximab combination therapy. Moreover, young gastroenterologists who have interest in IBD are more likely to prescribe both infliximab and adalimumab combination therapy than those who were either senior gastroenterologists with IBD interest or those with no IBD interest regardless of their age. In addition, gastroenterologists with IBD interest requested more anti-TNF serum drug concentrations and antidrug antibody level tests than those with no IBD interest. In conclusion, physicians with IBD interest or who are young, are more likely to follow IBD guidelines.

## Data Availability Statement

The original contributions presented in the study are included in the article/Supplementary Material, further inquiries can be directed to the corresponding author.

## Ethics Statement

The studies involving human participants were reviewed and approved by standing committee for coordination of health and medical research at the Ministry of Health in Kuwait (IRB 2020/1410). Written informed consent for participation was not required for this study in accordance with the national legislation and the institutional requirements.

## Author Contributions

IA: analysis and interpretation of data and drafting of the manuscript. GA and AA: acquisition of data and drafting of the manuscript. HA: drafting of the manuscript. MS: drafting of the manuscript, critical revision of the manuscript for important intellectual content, statistical analysis, and study supervision. All authors contributed to the article and approved the submitted version.

## Conflict of Interest

The authors declare that the research was conducted in the absence of any commercial or financial relationships that could be construed as a potential conflict of interest.

## Publisher's Note

All claims expressed in this article are solely those of the authors and do not necessarily represent those of their affiliated organizations, or those of the publisher, the editors and the reviewers. Any product that may be evaluated in this article, or claim that may be made by its manufacturer, is not guaranteed or endorsed by the publisher.
