# Supplementary material for: Gastroenterologists Adherence to Tumor Necrosis Factor Antagonist Combination Therapy in Inflammatory Bowel Disease
Source: Front Med (Lausanne). 2021 Sep 29;8:725512. doi: 10.3389/fmed.2021.725512 (PMC8511459; doi:10.3389/fmed.2021.725512)
Supplement: Supplementary file 1 [file Data_Sheet_1.PDF]

Supplementary Documents

Table (S1): Number and percentage of patients on monotherapy and combination therapy for infliximab stratified by gastroenterologist's age.

physicians	Monotherapy N (%)	Combination N (%)	Total N (%)
Senior gastroenterologists (age≥45, group D)			
A	10 (52.63%)	9 (47.36%)	19 (100%)
B	1 (50%)	1 (50%)	2 (100%)
C	0 (0%)	2 (100%)	2 (100%)
D	4 (40%)	6 (60%)	10 (100%)
A	1 (33.33%)	2 (66.66%)	3 (100%)
Total	16 (44.44%)	20 (55.55%)	36(100%)
gastroenterologists (age<45, group C)			
E	1 (20%)	4 (80%)	5 (100%)
F	0 (0%)	3 (100%)	3 (100%)

G	4 (44.44%)	5 (55.5%)	9 (100%)
H	2 (40%)	3 (60%)	5 (100%)
I	0 (0%)	7 (100%)	7 (100%)
J	4 (14.28%)	24 (85.71%)	28 (100%)
K	2 (9.09%)	20 (90.90%)	22 (100%)
L	1 (6.25%)	15 (93.75%)	16 (100%)
M	3 (50%)	3 (50%)	6 (100%)
Total	17 (16.8%)	84 (83%)	101(100%)

Table (S2): Number and percentage of patients on monotherapy and combination therapy for adalimumab stratified by gastroenterologist's age.

physicians	Monotherapy N (%)	Combination N (%)	Total N (%)
Senior gastroenterologists (age≥45, group D)			
A	17 (73.91%)	6 (26.08%)	23 (100%)
B	1 (100%)	0 (0%)	1 (100%)
C	0 (0%)	0 (0%)	0 (100%)
D	12 (80%)	3 (20%)	15 (100%)
A	6 (46.15%)	7 (53.84%)	13 (100%)
Total	36 (50%)	36 (50%)	52(100%)
gastroenterologists (age<45, group C)			
E	6 (54.54%)	5 (45.45%)	11 (100%)
F	1 (100%)	0 (0%)	1 (100%)
G	8 (72.72%)	3 (27.27%)	11 (100%)
H	3 (75%)	1 (25%)	4 (100%)

I	5 (71.42%)	2(28.57%)	7 (100%)
J	5 (21.73%)	18 (78.26%)	23 (100%)
K	3 (21.42%)	11 (78.57%)	14 (100%)
L	6 (28.57%)	15 (71.42%)	21 (100%)
M	4 (50%)	4 (50%)	8 (100%)
Total	41 (41%)	59 (59%)	100(100%)

Table (S3): Number and percentage of patients on monotherapy and combination therapy for infliximab stratified by inflammatory bowel disease (IBD) interest.

physicians	Monotherapy N (%)	Combination N (%)	Total N (%)
IBD interest (group A)			
A	1 (6.25%)	15 (93.75%)	16 (100%)
B	10 (52.63%)	9 (47.36%)	19 (100%)
C	4 (14.28%)	24 (85.71%)	28 (100%)
D	2 (9.09%)	20 (90.90%)	22 (100%)
Total	17 (20%)	68 (80%)	85 (100%)

Non-IBD interest (group B)			
E	0 (0%)	3 (100%)	3 (100%)
F	4 (44.44%)	(55.5%)5	9 (100%)
G	2 (40%)	3 (60%)	5 (100%)
H	0 (0%)	7 (100%)	7 (100%)
I	1 (50%)	1 (50%)	2 (100%)
J	0 (0%)	2 (100%)	2 (100%)
K	4 (40%)	6 (60%)	10 (100%)
L	1 (20%)	4 (80%)	5 (100%)
M	1 (33.33%)	2 (66.66%)	3 (100%)
N	3 (50%)	3 (50%)	6 (100%)
Total	16 (30.7%)	36 (69.2%)	52(100%)

Table (S4): Number and percentage of patients on monotherapy and combination therapy for adalimumab stratified by inflammatory bowel disease (IBD) interest.

IBD interest (group A)	Monotherapy N (%)	Combination N (%)	Total N (%)

A	6 (28.57%)	15 (71.42%)	21 (100%)
B	17 (73.91%)	6 (26.08%)	23 (100%)
C	5 (21.73%)	18 (78.26%)	23 (100%)
D	3 (21.42%)	11 (78.57%)	14 (100%)
Total	31 (38.27%)	50 (61.7%)	81(100%)
Non-IBD interest (group B)			
E	1 (100%)	0 (0%)	1 (100%)
F	8 (72.72%)	3 (27.27%)	11 (100%)
G	3 (75%)	1 (25)	4 (100%)
H	5 (71.42%)	2 (28.57%)	7 (100%)
I	1 (100%)	0 (0%)	1 (100%)
J	0 (0%)	0 (0%)	0 (100%)
K	12 (80%)	3 (20%)	15 (100%)
L	6 (54.54%)	5 (45.45%)	11 (100%)
M	6 (46.15%)	7 (53.84%)	13 (100%)
N	4 (50%)	4 (50%)	8 (100%)

Total	46 (64.78%)	25 (35.2%)	71(100%)

Table (S5): Number of anti-TNF serum concentration and antidrug antibody level tests performed by each physician and per infliximab and adalimumab

physicians	IFX tests<br>N (%)	ADL tests<br>N (%)	Total<br>N (%)
IBD interest (group A)			
A	19 (13.86%)	23 (15.1%)	42(14.53%)
B	28 (20.43%)	23 (15.1%)	51(17.65%)
C	22 (16.05%)	14 (9.21%)	36(12.46%)
D	16 (11.67%)	21 (13.81%)	37(12.80%)
Total	85 (51.2%)	81 (48.79%)	166 (100%)

**Non-IBD interest (group B)**			
**E**	**5 (3.64%)**	**11 (7.23%)**	**16 (5.54%)**
**F**	**3 (2.1%)**	**1 (0.65%)**	**4(1.38%)**
**G**	**9 (6.56%)**	**11 (7.23%)**	**20(6.92%)**
**H**	**5 (3.64%)**	**4 (2.63%)**	**9(3.11%)**
**I**	**7 (5.1%)**	**7 (4.60%)**	**14(4.84%)**
**J**	**2 (1.45%)**	**1 (0.65%)**	**3(1.04%)**
**K**	**2 (1.45%)**	**0 (0%)**	**2(0.69%)**
**L**	**10 (7.29%)**	**15 (9.86%)**	**25(8.65%)**
**M**	**3 (2.1%)**	**13 (8.55%)**	**16(5.54%)**
**N**	**6 (4.37%)**	**8 (5.26%)**	**14(4.84%)**
**Total**	**52 (42.27%)**	**71 (57.72%)**	**123 (100%)**

ADL: adalimumab, IFX: infliximab
